# Lactate: a rising star in tumors and inflammation

**DOI:** 10.3389/fimmu.2024.1496390

**Published:** 2024-11-26

**Authors:** Hui Liu, Mengmeng Pan, Mengxia Liu, Lin Zeng, Yumeng Li, Zhen Huang, Chunlei Guo, Hui Wang

**Affiliations:** Henan Key Laboratory of Immunology and Targeted Drug, Henan Collaborative Innovation Center of Molecular Diagnosis and Laboratory Medicine, School of Medical Technology, Xinxiang Medical University, Xinxiang, Henan, China

**Keywords:** lactate, tumor, inflammatory, diseases, therapy

## Abstract

Lactate has been traditionally regarded as a mere byproduct of glycolysis or metabolic waste. However, an increasing body of literature suggests its critical role in regulating various physiological and pathological processes. Lactate is generally associated with hypoxia, inflammation, viral infections, and tumors. It performs complex physiological roles by activating monocarboxylate transporter (MCT) or the G protein-coupled receptor GPR81 across the cell membrane. Lactate exerts immunosuppressive effects by regulating the functions of various immune cells (such as natural killer cells, T cells, dendritic cells, and monocytes) and its role in macrophage polarization and myeloid-derived suppressor cell (MDSC) differentiation in the tumor microenvironment. Lactic acid has also recently been found to increase the density of CD8^+^ T cells, thereby enhancing the antitumor immune response. Acute or chronic inflammatory diseases have opposite immune states in the inflammatory disease microenvironment. Factors such as cell types, transcriptional regulators, ionic mediators, and the microenvironment all contribute to the diverse functions lactate exhibits. Herein, we reviewed the pleiotropic effects of lactate on the regulation of various functions of immune cells in the tumor microenvironment and under inflammatory conditions, which may help to provide new insights and potential targets for the diagnosis and treatment of inflammatory diseases and malignancies.

## Introduction

Lactate is a metabolic end product of glycolysis and is mainly produced in the brain, skeletal muscle, intestine, and red blood cells. Under stress conditions, lactate is also produced in white blood cells, lungs, and viscera (1). The human body predominantly produces the L-isomer of lactate, facilitated by lactate dehydrogenase (LDH), an enzyme involved in both glycolysis and potential posttranscriptional gene regulation (2). Lactate connecting glycolysis to mitochondrial respiration in anaerobic conditions (3). In 1927, Otto Warburg noted that glycolysis in cancer cells under aerobic conditions can replace the normal aerobic cycle, resulting in a marked increase in lactate production, referred to as the “Warburg effect” (4). Tumor cells specifically consume glucose through Warburg metabolism, and tumor-infiltrating immune cells also rely on glucose. The altered metabolism of these immune cells within the tumor microenvironment contributes to the tumor cells’ ability to achieve immune escape (5). At the same time, the harsh metabolic microenvironment of tumors can also promote the Warburg effect in cells (6).

Now Lactate is understood to be produced and utilized under aerobic conditions as a signaling molecule. In the tumor microenvironment, lactate serves as a regulator of metabolic pathways, immune responses, and intercellular communication. Its significance extends beyond energy metabolism, making it an important target for understanding and potentially therapeutic intervention in various diseases, including cancer (7). At the systemic level, lactate metabolism is considered vital for at least three reasons (1): lactate serves as the primary energy source; (2) L-lactate is the primary isomer produced in humans; and (3) lactate is a signal molecule exhibiting autocrine, paracrine, and endocrine-like functions (3). The concepts of the “cell-to-cell lactate shuttle” and “intracellular lactate shuttle” describe the role of lactate in the transfer of oxidation and gluconogenic substrates, as well as in cell signaling.

Lactate triggers a series of intracellular signals that regulate various inflammatory responses in inflammatory diseases. Lactate levels were first identified as a marker of clinical outcome in patients with undifferentiated shock in 1964 (8). Since then, high lactate levels have been associated with ischemia, shock, diabetic ketoacidosis, trauma, liver dysfunction, and sepsis (8). In addition, lactate levels are associated with the level of inflammation. Given that the site of inflammation is in a hypoxic state (1% oxygen), the lactate level is often higher than normal (9). It has been established that the lactate concentration can increase to 15-17 mM in rheumatic synovial fluid (10), and in gingival sulcus liquid of patients with periodontitis, the levels can be as high as 19.69 ± 9.76 mM (11). In addition, Shapiro et al. reported that increased mortality in infected patients (such as pneumonia, severe sepsis or septic shock, bacteremia, etc.) was associated with increased lactate levels (12). Collectively, these results suggest that changes in lactic acid levels may significantly affect the inflammatory diseases. Lactate is excreted from tumor cells via monocarboxylate transporters (MCTs) after glucose metabolism, resulting in lactate accumulation in the tumor microenvironment (TME) and a decrease in pH (13). Under normal physiological conditions, the lactate concentration is approximately 1.5-3.0 mM, while the lactate concentration in the TME can reach 10-30 mM, and the pH can reach 6.0-6.5 (14–16). Interestingly, the tumor microenvironment creates an ecological environment that supports tumor growth rather than favoring antitumor immune monitoring, which is promoted to a certain extent by the accelerated metabolism of tumor cells and cancer-related fibroblasts (17). Increased metabolites in the TME, especially lactate, contribute to an immunosuppressive environment conducive to cancer cell growth and escape from the immune system (18). There is a growing consensus that lactate is not only an end product of glycolysis but also a key regulator of several signaling pathways in normal and tumor cells (3). For example, lactate has been shown to delay LPS-induced signaling pathways (19), while affecting several MAP kinases, NF-κB signaling, and the PI3K/AKT pathway (20–22). In this review, we focus on the immunomodulatory effects of lactate on tumors and inflammation, aiming to elucidate the mechanisms underlying the pathogenesis of diseases with similar acidic microenvironments and to provide novel insights and prospective targets for the treatment of related diseases.

## Lactate and tumors

### Lactate in the tumor microenvironment

The microenvironment of cancerous tissues is immunosuppressive and protumorigenic, whereas the microenvironment of tissues affected by chronic inflammatory diseases is proinflammatory and antitumor. Despite these opposing immune states, the metabolic states in the tissue microenvironments of cancer and inflammatory diseases are similar: both are hypoxic, with elevated levels of lactate and other metabolic byproducts and reduced levels of nutrients [7]. The TME comprises the surrounding mesenchymal and immune cells, the extracellular matrix, and metabolites and signaling molecules in the intercellular space (23). Current evidence suggests that the TME plays an active role in regulating key features of cancer, including energy metabolism, protumor growth, angiogenesis, invasion and metastasis, and immune evasion (24). Many cells reportedly contribute to the establishment of the TME (25). In this environment, the balance of normal cells and tissues is disrupted, resulting in the production of pro-/antitumor growth factors, extracellular vesicles, cytokines, extracellular matrix (ECM) proteins, and ECM remodeling enzymes that trigger a shift of surrounding cells to a more tumor-friendly immune response (24). In addition, the TME has anoxic or semianoxic properties (26).

**Figure 1:**
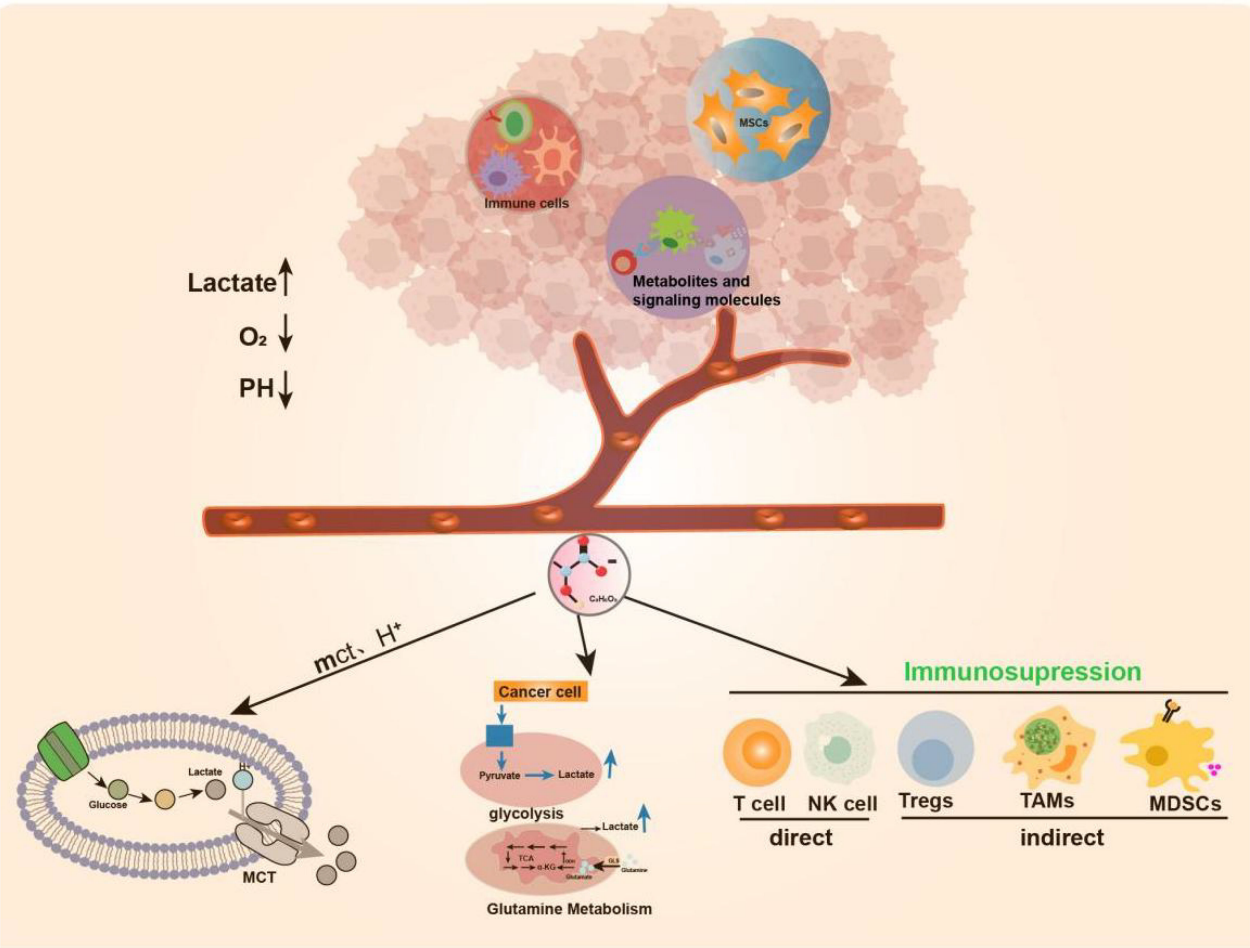
Production of Lactate in TME. TME includes surrounding mesenchymal and immune cells, extracellular matrix, metabolites and signaling molecules in the intercellular space.TME has anoxic or semi-anoxic properties, and tumor cells metabolize glucose or glutamine to produce a large amount of lactic acid, Lactate is co-transported by monocarboxylate transporters (MCTs), along with protons (H^+^), resulting in the accumulation of lactic acid and acidification of the tumor microenvironment.

Tumor cell metabolism produces a large amount of lactate, Lactate is co-transported by monocarboxylate transporters (MCTs), along with protons (H^+^), resulting in the accumulation of lactate and acidification of the tumor microenvironment (27). New research has revealed that the elevated activity of glycolytic and glutamine enzymatic pathways in cancer cells is the foremost cause of lactate accumulation in the TME. Additionally, lactate buildup in the TME impedes the immune system’s ability to fight against tumors (4, 28–30). More importantly, lactate is a major disruptor of TME immune function. On the one hand, it can directly mediate immunosuppressive effects on other cells (NK cells and T cells) by blocking the function of cytotoxic, motility, or transcription factors of immune cells. On the other hand, it can indirectly exert its immunosuppressive function by inducing immunosuppressive cells such as Tregs, TAMs, and MDSCs (31) (**Figure 1**).

### Lactate in immune escape of tumors

Cancer is a multifaceted disease characterized by the buildup of mutated genetic material, constant apoptosis of healthy cells, and uncontrolled growth and spread of cancerous cells (32). The “immune escape” of tumors is considered one of the primary reasons behind their development. Rich literature substantiates that the metabolism of tumors is tightly correlated with the body’s immunity. Tumor cells take up glucose under hypoxic or “Warburg effect” conditions and release lactate, an essential metabolic byproduct, to alter their microenvironment. The massive accumulation of lactate exerts tumor immunosuppressive effects by regulating and affecting the functions of various immune cells and the release of cytokines. There is growing evidence that immune surveillance deficiencies promote immune escape from tumors, thus favoring tumor cell development and survival (33, 34). First, lactate affects immune cell cytotoxicity, motility, and transcription factor expression, leading to inhibition of the function of T-cells and NK cells. According to Brand et al., high lactate levels serve as strong inhibitors of T-cell and NK cell function and survival, thus facilitating the immune escape of tumors (24). For example, lactate can exert a protumor immune escape effect by directly inhibiting the lysis capacity of natural killer (NK) cells or inducing their apoptosis (35–37). In T cells, lactate can regulate CD4^+^ T-cell polarization, inhibit antitumor Th1 cells, promote Treg differentiation, and maintain the progression of prostate cancer (PC) through the TLR8/miR21 axis (38). Lactate produced by highly glucose-dependent tumors can also impair the infiltration and function of cytotoxic CD8^+^ T cells, thereby preventing immune surveillance (39, 40). Furthermore, studies have shown that the production of type I interferon (IFN-γ) in both T cells and NK cells is inhibited by lactate, thereby limiting immune cell activation (24). Therefore, lactate served as a key metabolite of glycolysis-mediated inhibition of RIG-I like receptors (RLR) signaling, can reduce host defense against viral infection and cancer immunosurveillance by inhibiting the production of IFN-γ (41). These findings suggest that lactate can play an essential role in tumor development by altering the behavior of immune cells (e.g., T cells and NK cells) in theTME (42).

**Figure 2:**
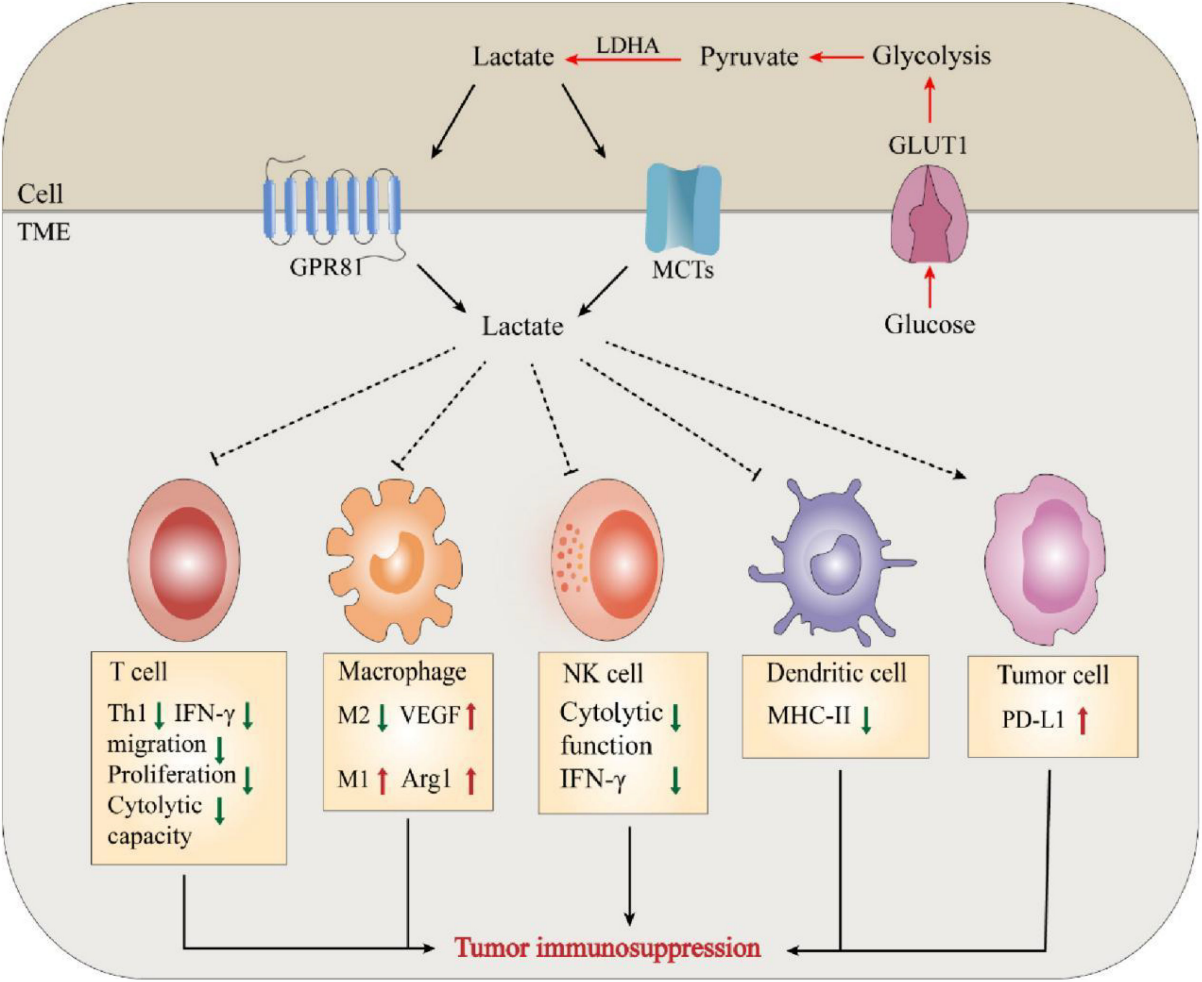
Lactate mediates tumor immunosuppression in TME. The massive accumulation of lactate exerts tumor immunosuppressive effects by regulating and affecting the functions of various immune cells and the release of cytokines.

Lactate is also involved in immune escape by altering several immune infiltrating cells. Like macrophages in normal tissues and organs, TAMs play a critical role in maintaining tumor growth and homeostasis (43). Macrophages can be classified as M1/M2-type macrophages depending on their source of energy (glycolysis or oxidative metabolism) (44). Zhang et al. documented a novel epistatic modification mechanism that stimulates histone lactonization by lactate produced during hypoxia (45). By increasing histone lactonization, lactate can trigger the polarization of macrophages from the M1 phenotype, which is cytotoxic and inflammatory, to the M2 phenotype, which is more tumor friendly, thereby suppressing the immune response within the TME (44, 46). Moreover, Colegio et al. reported that lactate produced by cancer cells induces increased expression of arginase 1 (Arg1) and vascular endothelial growth factor (VEGF) derived from tumor-associated macrophages (TAMs), thereby supporting tumor growth (47). In addition, lactate immunosuppression mechanisms include driving immune escape by increasing the infiltration of suppressor cells in the tumor environment, such as myeloid-derived suppressor cells (MDSCs) (48). Tumor-derived lactate can inhibit NK cell function by increasing the level of MDSCs, thus playing an immunosuppressive role in suppressing the natural immune response against tumor development (49) (**Figure 2**).

The exchange of lactate between tumor cells or mesenchymal cells and the TME is mediated by the corresponding lactate transporters. Monocarboxylate transporters (MCTs) belong to the SLC16 family of the solute carrier (SLC) superfamily, and the SLC superfamily consists of over 400 protein membrane transporters, four allozymes (MCT1-4) are involved in lactate transport (50, 51). In this regard, cytotoxic T cells (CTLs) are the main effector cells of antitumor immunity, and the pathway by which lactate is produced by glycolysis in the TME is usually mediated by MCTs (52, 53). However, high lactate levels in the TME inhibit this process, and consequently, intracellular lactate inhibits the proliferation of CTLs and the production of cytokines, perforin, and granzyme B, resulting in a decrease in cytotoxic activity of 50% (54). Lactate exerts its protumor effects by binding to the cell surface receptor GPR81. Activation of GPR81 ultimately leads to the promotion of vasculogenesis, evasion of the immune system, and resistance to chemotherapy (55). Recent studies have shown that lactate activates GPR81 in tumor cells, which results in elevated PD-L1 production and reduced IFN-γ expression, highlighting the role of autocrine mechanisms (56). In addition, the above autocrine mechanism was complemented by the paracrine mechanism reported by Brown et al. in a mouse model of constitutive breast cancer (MMTV-PyMT-Tg) (46). They showed that GPR81 is upregulated in breast cancer and can exert an autocrine effect through tumor cell-derived lactate, thereby promoting tumor growth. Tumor cell-derived lactate can also exert protumorigenic effects by activating GPR81 in dendritic cells and blocking the presentation of MHCII cell surface tumor-specific antigens. These findings suggest that lactate promotes tumor activity through the GPR81 signaling pathway during tumor development through autocrine or paracrine secretion (57).

### Lactate in tumor inflammation and angiogenesis

**Figure 3:**
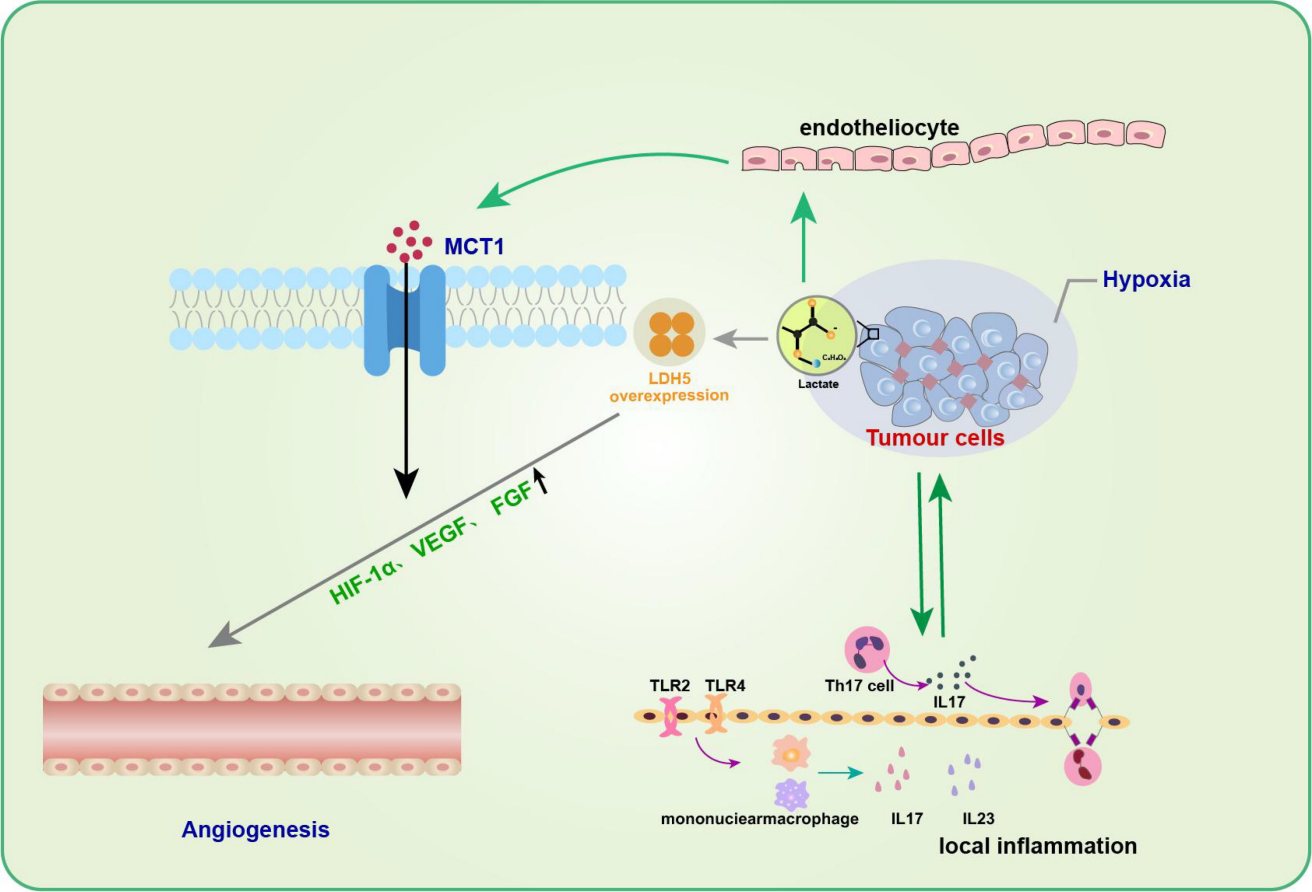
Lactate in tumor inflammation and angiogenesis. Lactic acid promotes angiogenesis of tumor cells by affecting endothelial cells. The entry of lactic acid into the tissue stroma by tumor cells can be facilitated by endothelial cells via MCT1 to inhibit HIF-1α degradation of non-hypoxic endothelial cells and significantly up-regulate endothelial cell production of VEGF and fibroblast growth factor (FGF), thus promoting angiogenesis. In addition to promoting neovascularization, the role of lactic acid in promoting tumor development is often associated with inflammatory responses.Th17 cells in the tumor microenvironment gradually increase and promote the secretion of cytokine IL-17 during tumor development, which can up-regulate the production of a variety of pro-inflammatory cytokines and pro-angiogenic factors, and promote tumor development.

Neovascularization is an essential biological process in tumor growth, infiltration, and metastasis. Many studies have substantiated the positive role of lactate in promoting angiogenesis, reducing muscle atrophy, and accelerating wound healing during trauma healing (58, 59). Kolev et al. retrospectively analyzed 152 patients with different stages of gastric cancer and found a significant correlation between LDH5 overexpression and increased levels of HIF-1α and VEGF, indirectly suggesting that lactate metabolism contributes to angiogenesis (60). Moreover, lactate can promote angiogenesis in tumor cells by affecting endothelial cells (61, 62). Sonveaux et al. found that lactate excreted by tumor cells into the tissue interstitium could be taken up by endothelial cells with the help of MCT1, which inhibited the degradation of HIF-1α in nonhypoxic endothelial cells and significantly upregulated endothelial cell production of VEGF and fibroblast growth factor (FGF), thus promoting angiogenesis (61) (**Figure 3**). In contrast, the application of MCT1 inhibitors reversed these effects, further confirming the proangiogenic effect of lactate. These studies highlight the need to further investigate the association between lactate metabolism and angiogenesis and its underlying mechanisms.

In addition to facilitating neovascularization, the role of lactate in promoting tumor development is often associated with an inflammatory response. Heinemeyer et al. showed that Th17 cells in theTME gradually increase and promote the secretion of the cytokine IL-17 during tumor development (63), which can upregulate the production of various proinflammatory cytokines and proangiogenic factors to promote tumor development (64, 65). IL-23, a proinflammatory cytokine, has also been shown to be expressed excessively in and around tumor cells, thereby inducing topical inflammation and promoting tumor growth. Shime et al. further found that in TLR2 and TLR4 agonist-stimulated monocytes/macrophages and tumor-infiltrating immune cells, tumor cells could secrete lactate to activate the IL-23/IL-17 pathway and promote tumorigenesis and growth by promoting angiogenesis and triggering a local inflammatory response (66).

### Role of lactate in invasive metastasis of tumors

Numerous studies on lactate in different types of tumors, including colorectal cancer (35), cancer of the cervix (67), head and neck cancer (68, 69), and stomach cancer (70), have confirmed that lactate levels strongly correlate with tumor invasion and metastasis. Harmon et al. showed that lactate-mediated activation of the TME could be induced by liver-resident NK cell apoptosis, which leads to the survival of metastatic cancer cells in the liver (35). Further research has shown that lactate produced by CRLM (Coloretal Liver Metabases) can lower the pH in the tumor microenvironment, causing liver NK cells migrating to the tumor to lose their ability to regulate intracellular pH. This, in turn, results in mitochondrial stress and apoptosis in NK cells, ultimately benefiting the survival of metastatic cancer cells and promoting tumor metastasis. Rizwan et al. also reported that LDHA knockdown delayed tumor metastasis and improved overall survival in a mouse model of breast cancer (71). Interestingly, Hirschhaeuser et al. noted that tumor metastasis is promoted by lactate-induced secretion of hyaluronic acid by tumor-associated fibroblasts, which creates an environment conducive to migration and thus promotes tumor metastasis (72). Notably, evidence from mouse cancer models suggests that tumor metastasis can be inhibited by neutralizing acidity with oral buffers (73, 74). These studies suggested that lactate is closely associated with tumor invasion and metastasis.

However, the exact mechanism by which lactate is involved in tumor metastasis is not fully understood. Baumann et al. discovered that lactate could stimulate transforming growth factor-B2 (TGFB2) expression in glioma cells. TGFB2 is a critical regulator of glioma cell migration and is likely one of the significant mechanisms underlying glioma metastasis (75). In addition, the overexpression of HIF-1α in tumor cells leads to metabolic reprogramming, resulting in the Warburg effect, which results in the production of large amounts of lactate (76). HIF-1α has also been associated with breast cancer growth and metastasis and poor prognosis and aggressiveness. These findings offer a new approach for preventing breast cancer metastasis (77, 78). Notably, cells in a high-lactate environment exhibit rapid increases in the mRNA and protein expression of the transporter protein MCT1 (79). In addition, high levels of MCT1 expression have been associated with cancer invasion in non-small cell lung cancer and melanoma (80, 81). Similarly, Zhao et al. proposed that the molecular mechanism underlying osteosarcoma cell migration involves enhanced NF-κB signaling and is facilitated by MCT1 (82). Similar conclusions were obtained in a single test on cervical and mammary cancer cells (83). To conclude, future research should investigate the precise mechanisms through which lactate regulates tumor invasion and metastasis in various types of tumors due to the heterogeneity and complexity of tumor metabolism.

### Lactate in oncology treatment

Lactate plays dual roles as a fuel for metabolism and a signaling molecule in tumors (4). In addition, lactate is necessary for the growth and development of tumor cells (84). Therefore, targeting the aberrant production of lactate in tumor cells, a key factor in cancer genesis and progression, has become a promising approach for cancer therapy (85) (**Table 1**).

**Table 1:** Lactate in oncology treatment.

Target	Drug	Mechanism of action
Metabolic intervention in cancer		
**LDHA**	GNE-140	Inhibit Arg1
**PD1**	Nivolumab	Bind to PD1, blocking its interaction with PDL1 and PDL2
	Pembrolizumab	
	Pidilizumab	
**PDL1**	BMS935559	Bind to PDL1, blocking its interactions with both PD1 and B7.1 receptors
	MPDL3280A	
	MSB0010718C	
**MCT1**	AZ3965	Inhibit SLC16A1 and SLC1647
	AR-C155858	Inhibit SLC16A1 and SLC1647
	SR1380	Inhibit SLC16A1 and SLC1647
**CTLA4**	Ipilimumab	Bind to CTLA4, blocking the inhibitory signal, which enables CTLs to kill cancer cells
**PAPR**	Olaparib	Specific DNA repair defects
**HIF**	PT2399	Inhibit HIF
**mTORC1/2**	AZD2014	Suppress mTOR signaling pathway
**VEGF**	Sorafenib	Block the tyrosine kinase activity
**ATR**	AZD6738	Modulate ATR/ATM/DNA-PK signaling
**miR21**	ADM-21	Inhibit miR-21
**PI3K/AKT**	Erlotinib	Induce autophagy through PI3K/AKT/mTOR-mediated pathway
	Cetuximab	

Lactate metabolism has become an attractive target for enhancing cancer immunotherapy (86, 87). PI3K-Akt-mTOR-HIF axis was firstly used to inhibit lactate production by indirect targeting of glycolysis, and Akt inhibitors are being explored in patients with advanced or metastatic solid tumors in combination with a PARP inhibitor and anti-PD-L1 (NCT03772561) (88). Recent studies showed that targeting HIF-1α could increase CD8^+^T cell infiltration and support anti-PD-1/PD-L1 treatment as effectively as CTLA-4 blockade (89, 90). Inhibiting or eliminating the m6A demethylase ALK-BH5 leads to a notable decrease in lactate levels and the recruitment of Treg cells and MDSCs within the TME during anti-PD-1 therapy in mouse models of melanoma and colorectal cancer (91).

Lactate and lactic acid on the effectiveness of tumor vaccines were also studied. Feng et al. found that PC7A nano-tumor vaccine in lactate solution could significantly improve anti-tumor efficacy using an MC38 mouse tumor model (40). Lactic acid can augment the immunogenicity of whole UV-irradiated tumor cell vaccines by promoting dendritic cell (DC) maturation, aggregation and phagocytosis in mouse xenograft models (92). Furthermore, the injection of lactic acid-stimulated tumor vaccines could significantly reduce the number of CD11b^+^Gr1^+^MDSCs in tumor tissues, which plays a crucial role in immune evasion, tumor occurrence, and development (93). In the latest research, NaHCo3NPs nanoparticles could alleviate immune suppression and pyroptosis induced immune activation through acid neutralization, exhibiting enhanced anti-tumor immune efficiency by inhibiting primary/distal tumor growth and metastasis (94).

It is known that lactate dehydrogenases play a crucial role in lactate production. LDH enzymes are a subset of metabolic enzymes that facilitate the change from pyruvate to lactate and are equally crucial in cancer metabolism (95). Hence, targeting lactate production has emerged as a promising strategy for cancer therapy, and inhibitors targeting LDH enzymes are being extensively studied for their effectiveness in cancer treatment (96, 97) (**Table 1**). For example, GNE-140, a small molecule inhibitor of LDHA, has been shown to effectively inhibit the development of B16 melanoma in mice and human adenocarcinoma and pancreatic cancer *in vitro*, and its drug activity depends on cell metabolic activity (98, 99). Fang A et al. tested a prospective new compound (compound 11) in the MG-63 osteosarcoma cell line (100). The results showed that compound 11 could inhibit LDHA by reducing lactate production and causing extrinsic metabolic acidification (99). Given the complexity of lactate metabolism in the development of different tumors, despite the numerous effective LDH enzyme inhibitors reported, very few are currently available for clinical use. Therefore, further research is needed to optimize existing compounds and develop new lactate dehydrogenase inhibitors to improve cancer therapy.

In addition, lactate exchange between various tumor cells, mesenchymal cells, and the TME, is one of the necessary pathways for tumor cell nutrient depletion. Therefore, targeting the key molecules associated with lactate transport is an effective therapeutic approach for treating tumors. Among them, targeting MCTs may significantly impact intercellular lactate exchange (101, 102). Inhibition of MCT1 specifically impairs the influx of intercellular lactate, forcing metabolic conversion of tumor cells to aerobic glycolysis. This indirectly leads to the death of anoxic cancer cells due to glucose deprivation (103, 104). Common MCT inhibitors include AstraZeneca’s AZ3965 compound, as well as AR-C155858 and SR1380 [95,96]. These inhibitors specifically or nonspecifically target MCT1 and/or MCT2, leading to impairment of cancer cell survival and proliferation and ultimately exerting antitumor effects (105). In addition to MCT1, blocking lactate transport between diverse cell groups by downregulating the MCT4 gene may provide an efficient treatment strategy (106, 107). For example, A recent study has shown that inhibiting MCT4 could enhance the therapeutic efficacy of anti-PD-1 therapy and the combination treatment of MCT4 inhibitors and anti-PD-L1 therapy exhibited beneficial effects in 3D colorectal cancer sphere models (108). AstraZeneca’s MCT4 inhibitor AZ93 can not only target MCT4 but also reduce the propagation of various cancer cell lineages in which MCT1 is repressed (109).

Interestingly, clinical and retrospective analysis revealed that MCT4 could play a compensatory role when MCT1 is inhibited (101, 105). Therefore, it is highly conceivable that under hypoxic conditions, simultaneously inhibiting MCT1 and MCT4 can disrupt tumor growth. Dual inhibitors targeting both MCT1 and MCT4 have exhibited promising results as potential antitumor agents (110–112). Pilon-Thoma et al. reported that neutralizing the tumor pH with bicarbonate inhibited tumor growth in mice (113). In conclusion, the future of antitumor therapy may be more dedicated to combining various antitumor tools, such as chemotherapy, radiotherapy, and inhibitors, to observe their additional or synergistic effects to achieve better efficacy in oncology treatment.

## Lactate and inflammation

### Lactate and acute inflammation

**Figure 4:**
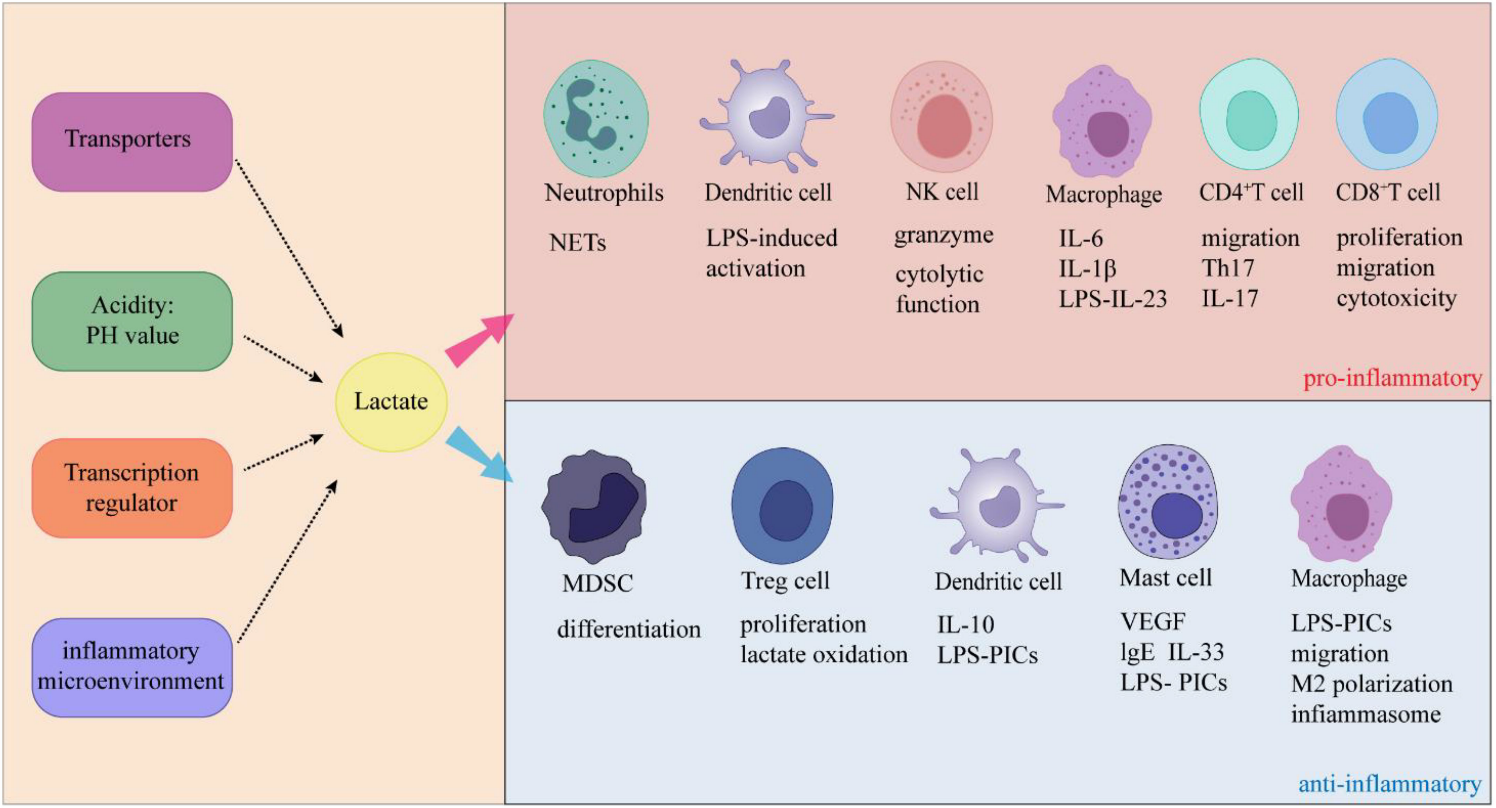
Lactate exerts dual effects on inflammation through its specific effects on immune cells.

In recent years, studies reported that elevated lactate levels in inflammatory diseases due to impaired production or clearance of lactate, which affects immune cell function (114). The cellular entry of lactate is dependent on MCT-1 and SLC5A12 (41, 115). Once lactate and its associated H^+^ ions enter the cell, they typically function as negative feedback regulators of glycolytic ATP production (114). In general, glycolysis supplies fuel to inflammatory cells, while oxidative phosphorylation is the origin of energy for anti-inflammatory regulatory cells (116), suggesting that lactate-mediated glycolysis may inhibit the function of inflammatory cells and promote regulatory function (**Figure 4**). Below, we discuss how lactate affects immune cells and thus suppresses inflammation.

Specific receptor signaling cascades in monocytes and macrophages have been demonstrated to be suppressed by lactate (114). For example, lactate delays the phosphorylation of protein kinase B (AKT), inhibits the degradation of IκB-α inhibitors, and promotes the accumulation of NF-κB in the nucleus, thereby inhibiting LPS receptor signaling (19, 117). Lactate can also stimulate GPR81-induced inhibitory signals that impair TLR-4-mediated inflammasome assembly, among other effects (41, 117). In addition, lactate also suppresses the activation of dendritic cells and reduces the production of cytokines induced by LPS (118, 119). Lactate can activate the GPR81 receptor and enhance Th2 activation on dendritic cells, leading to a decrease in antigen presentation and cytokine production and a reduction in cAMP activation (46, 120). This finding suggested that lactate inhibits the activation of mononuclear cells and suppresses cell-mediated inflammation.

Lactate can inhibit inflammation mediated by mast cells. On the one hand, lactate can inhibit IL-33-induced mast cell activation both *in vitro* and *in vivo* (115). Abebayehu et al. pointed out that lactate can enhance the expression of HIF1-α in a MCT-1 and pH dependent manner (121). After activating IL-33 and LPS, lactate can amplify inflammatory signals by inhibiting the expression of miR-155-5p (122, 123). On the contrary, mimetics of miR-155-5p can reverse the inhibitory effect of lactate, indicating that lactate may maintain the negative feedback pathway by inhibiting the expression of miR-155-5p (123). On the other hand, lactate can affect the function of mast cells by inhibiting calcium mobilization, degranulation, and release of cytokines and chemokines under the influence of MAS-related GPR coupled receptor X2 (mrgprx2) (124).

Like mast cells, lactate also inhibits regulatory T cells. Studies have shown that regulatory T cells can absorb and metabolize lactate to maintain their suppressive function under high lactate conditions (125). Several studies have reported that lactate reduces the IL-1β-induced transcription of key proinflammatory cytokines, such as IL-1β, IL-6, CCL2, and Pghs2, via GPR81 (2). Lactate produced during labor can also eliminate or reduce inflammation in a negative feedback manner via GPR81 in the uterus (126). Interestingly, lactate reportedly suppresses proinflammatory signaling pathways via a mechanism that does not involve GPR81 (127). The transcription of the proinflammatory genes IL-1B, IL-6, IL-12B, and CD40 was downregulated after lactate treatment in the bone marrow mesenchymal stem cells (BMMS) of GPR81-deficient mice (127). Furthermore, lactate has been shown to impair metabolic reprogramming following macrophage activation in a GPR81-independent manner (127), which has also been associated with inhibiting the response to proinflammatory cytokines (128). These findings suggest that GPR81 may play an essential role in the depression of inflammation by lactate, but whether this anti-inflammatory effect depends on GPR81 in different pathophysiological environments remains to be further explored.

### Lactate and chronic inflammation

Unlike acute inflammation, the most dominant feature of chronic inflammation is the infiltration of mononuclear cells such as lymphocytes, macrophages, and plasma cells (129). Lactate promotes immune cell inflammation during chronic inflammation (**Figure 3**). Below, we discuss the impacts of lactate on immune cells during chronic inflammation.

Lactate has been found to inhibit the cytolytic function of NK cells and reduce cell proliferation, motility, cytolytic activity, and the secretion of degranulation and inflammatory mediators in CD8^+^ T cells (54, 130, 131). These effects of lactate are attributed to its dependence on MCT-1, which inhibits the activity of protein kinases (130, 132). Interestingly, the effects of lactate on CD8^+^ T cells are not solely dependent on the inhibition of glycolysis. Lactate inhibits the locomotion of CD8^+^ T cells while decreasing cytolytic capacity by increasing inflammatory cytokine production, prolonging chronic inflammation (132, 133). Furthermore, CD8^+^ T cells reportedly contribute to rheumatoid arthritis (RA) by releasing proinflammatory and cytolytic mediators in the synovial membrane (SM) microenvironment lacking oxygen and nutrients (134).

Lactate and lactic acid have distinct effects on CD4^+^ T cells. Lactate inhibits the movement of CD4^+^ T cells, while lactic acid promotes the differentiation of naive CD4^+^ T cells into proinflammatory Th17 cells (114). Unlike CD8^+^ T cells, CD4^+^ T cells rely on the expression of SLC5A12 rather than MCT-1. This difference in transporter dependence may explain the opposing effects of lactate and lactic acid on immune cells (132). By acting through SLC5A12, lactate not only hinders the production of glycolytic energy but also boosts oxidative stress, thereby promoting the translocation of pyruvate kinase to the nucleus. In the nucleus, pyruvate kinase phosphorylates signal-transducing nuclear transduction activators (STAT3/1) and triggers RORγt-dependent IL-17 transcription (133). Pucino et al. reported that lactate driven by SLC5A12 leads to the reprogramming of cellular metabolism and facilitates IL-17 production via PKM2/STAT3 signaling, thereby promoting the inflammatory response of CD4^+^ T cells in patients with rheumatoid arthritis (133). These findings have significant implications for understanding the role of lactate in inflammatory disorders such as synovial membrane infection in rheumatoid arthritis patients. Interestingly, unlike that of CD8^+^ T cells, the inhibition of CD4^+^ T-cell motility by sodium lactate is dependent on interference with glycolysis. These findings suggest a complex mechanism by which lactate or sodium lactate modulates T-cell motility, and further studies are needed to investigate its pathological significance and molecular mechanism. Current evidence suggests that sodium lactate induces the conversion of CD4^+^ T cells into a Th17 subpopulation that generates large quantities of the proinflammatory cytokine IL-17 [69]. IL-17, a proinflammatory cytokine secreted by Th17 cells, also plays a key role in the pathogenesis of periodontitis (135). One study revealed that the concentrations of lactate and lactate dehydrogenase in the gingival crevicular fluid of chronic periodontitis patients were also elevated due to the hypoxic periodontal microenvironment. After undergoing nonsurgical treatment, patients with periodontitis exhibit reductions in lactate levels, IL-17 concentrations, and Th17 cell counts [11]. This observation raises the question of whether there is a link between increased lactate levels and elevated IL-17 levels in periodontitis, although further research is needed to confirm this association.

In addition, sodium lactate could increase the secretion of LPS-stimulated matrix metalloproteinases (MMP) -1, IL-1β, and IL-6 of U937 histiocytes by enhancing AP-1 and NF-κB transcriptional activities. Lactate could boost TLR4 signaling and NF-κB mediated gene transcription in macrophages via MCTs (136, 137). It has also been reported that monocyte differentiation is capable of increasing inflammatory (M1) and regulatory (M2) mediators when lactate is present in conjunction with granulocyte-macrophage colony-stimulating factor or in adenocarcinoma-conditioned media (elevated lactate and many other mediators), consistent with a TAM phenotype (138, 139). Further experiments revealed that the combination of GM-CSF and lactate drives the production and depletion of IL-6-dependent macrophage colony-stimulating factor (M-CSF), promoting an inflammatory feedforward loop that exacerbates inflammation.

### The possible mechanisms of lactate regulating inflammation

In the previous section, we discussed that the differential effects of lactate on CD4^+^ T cells and CD8^+^ T cells could be attributed to the selective expression of these cells to SCL5A12 and MCT-1, respectively. Lactate can inhibit the proliferation, degranulation, motility, cytolytic activity, and secretion of inflammatory mediators by CD8^+^ T cells. Lactate mediated the switch from CD4^+^ T cells to Th17 cells, promoting inflammation. It is highly conceivable that differences in acidity and concentration play a role in this mechanism. Several studies have reported that the effect of lactate is pH dependent (140). The role of acidity was maintained in dendritic cells, where its inhibitory effect was reversed by modulating the pH to 7.4 at a lactate concentration of 10 mM but was less effective at lactate concentrations above 10 mM (118). This finding suggested that the lactate concentration can also alter the inhibitory effect of the same transporter. Another mechanism may involve the effect of lactate on transcriptional regulators such as HIF-1α and HIF-2α. Lactate promotes HIF-1α activity while inhibiting glycolysis and the production of inflammatory cytokines (47, 141). Lactate has been shown to selectively induce HIF-1α-dependent transcription of VEGF while also promoting Th17 polarization. It is widely thought that lactate controls HIFs through multiple levels of regulation, including transcriptional and posttranslational regulation. This allows lactate and associated H^+^ to influence the function of these important transcription factors in the regulation of inflammation and angiogenesis (141–143). This phenomenon may be attributed to the fact that lactate can enhance the biological responses of Th17, Th2, and M2 cells in distinct inflammatory microenvironments (133). Although the exact mechanism remains unclear, lactate has been demonstrated to stimulate Th17 differentiation and induce IL-23 production. As we mentioned earlier, Th17 and IL-23 are significant in inflammation. These proposed mechanisms suggest that future research should elucidate how lactate affects various types of immune cells.

## Prospect

Herein, we investigated the role of lactate in tumors and inflammation. On the one hand, lactate can act as a metabolite that promotes tumor growth and development; on the other hand, the immunoregulatory effect of lactate has dual roles in acute and chronic inflammation.

Lactate promotes tumor growth, immune escape, tumor inflammation, angiogenesis, and tumor metastasis by regulating the function of various immune cells. We discussed the dual role of lactate as both a metabolic fuel and a signaling molecule. Currently, cancer continues to be a disease with a high mortality rate. The presence of lactate can promote tumor growth and metastasis and plays an essential role in tumor progression. However, the currently available drugs are far from meeting our needs. We should dedicate more resources to the development of therapeutic drugs in the future, as inhibiting the tumor-promoting effect of lactate holds promise for the recovery of cancer patients.

Lactate plays a beneficial role during acute inflammation by acting as a negative feedback regulator that inhibits the function of immune cells, thus reducing inflammation. However, elevated lactate levels can prolong the course of inflammation during chronic inflammation, indicating a different role for lactate in this context. However, the specific effects of cells, transcriptional regulators, H^+^ mediators, and the microenvironment on lactate remain largely unclear and warrant further study.

In summary, although it has been established that lactate can have both positive and negative effects on our bodies, more research is warranted. In this respect, future studies should focus on two areas: 1) developing effective pharmaceuticals to inhibit lactate and its tumor-promoting effects and 2) investigating how lactate influences the development of different cell types and the role of the microenvironment in shaping these responses.
